# Murine Model of Radiation Dermatitis with Experimental Wound and Effects of Genistein

**DOI:** 10.3390/ijms27115019

**Published:** 2026-06-02

**Authors:** Ernest O. N. Phillips, Amal Alzahrani, W. Bradley Rittase, John E. Slaven, Donald C. Aduba, Sandhya Xavier, Ji-an Wang, Evelyn C. Hays, Duane Craig, Georgia E. Streett, Leonard Sperling, Sang-Ho Lee, Helena B. Pasieka, Thomas N. Darling, Regina M. Day

**Affiliations:** 1 Department of Medicine, Division of Dermatology USUHS, Uniformed Services University of the Health Sciences, Bethesda, MD 20814, USA; ernest.phillips.ctr@usuhs.edu (E.O.N.P.); donald.aduba.ctr@usuhs.edu (D.C.A.J.); sandhya.xavier.ctr@usuhs.edu (S.X.); jwang0651@gmail.com (J.-a.W.); leonard.sperling@usuhs.edu (L.S.); helena.pasieka@usuhs.edu (H.B.P.); thomas.darling@usuhs.edu (T.N.D.); 2 Department of Pharmacology and Molecular Therapeutics, Uniformed Services University of the Health Sciences, 4301 Jones Bridge Rd., Bethesda, MD 20814, USA; amal.alzahrani.ctr@usuhs.edu (A.A.); w-bradley.rittase.ctr@usuhs.edu (W.B.R.); john.slaven.ctr@usuhs.edu (J.E.S.); e.clare@verizon.net (E.C.H.); duane.craig.ctr@usuhs.edu (D.C.); georgiastreett@gmail.com (G.E.S.); 3 Pathology Department, Research Services, Naval Medical Research Command, Silver Spring, MD 20910, USA; sang.h.lee52.mil@health.mil

**Keywords:** dermatitis, ionizing radiation, accelerated senescence, p21/waf1, hair follicle stem cells, genistein

## Abstract

Cutaneous Radiation Injuries (CRIs) and wounds within an area of radiation exposure (combined injury, CI) are a significant concern for nuclear accidents and radiation combat/terrorist events. CRIs and CI present unique clinical challenges, and effective countermeasures are urgently needed. Here we describe a murine model of CRI and CI in C57BL/6 mice using 16.9 Gy thoracic X-ray irradiation (5.3 Gy/min, 160 kV) ± experimental wound administered immediately. Wound repair and radiation-induced dermatitis were assessed after irradiation. Our previous studies showed that genistein (200 mg/kg, s.c.), administered 24 h prior to irradiation prevented radiation injuries in two murine models. We investigated the effects of genistein in the CI model. Macroscopic and histological analyses showed that radiation significantly delayed wound closure, although wounds did not significantly alter the progression of radiation dermatitis. Genistein improved the early rate of wound closure and significantly reduced dermatitis in mice. Histological analysis showed that genistein improved skin structure and reduced inflammation and fibrosis. Immunohistochemistry showed that genistein attenuated radiation-induced cyclin-dependent kinase inhibitor 1 (p21/waf1) and α-smooth muscle actin and preserved K15 positive skin adult stem cells. These findings suggest that genistein may be an effective prophylactic against CRIs and CI.

## 1. Introduction

The global growth in nuclear power generation and proliferation of nuclear weapons has increased the population’s risks for accidental radiation exposures [1,2]. Additionally, there is a parallel growth of radiotherapy usage as part of curative therapy for cancer [3]. Exposure to partial- or total-body irradiation (TBI) is potentially lethal through the induction of acute radiation syndrome (ARS) [4,5]. Cutaneous Radiation Injuries (CRIs) are a major contributor to ARS and morbidity following high-dose radiation exposure and are a significant concern for nuclear accidents and radiation-related combat or terrorist events [6,7,8,9,10,11,12]. Non-healing radiation dermatitis is a significant medical challenge in patients undergoing radiotherapy and is considered a factor that can affect quality of life after radiotherapy [12,13,14,15].

Besides potential CRIs, studies have estimated that in a radiation mass casualty or combat/terrorist scenario, 65–70% of individuals with radiation exposures will have other injuries, including wounds [16], a condition termed Combined Injury (CI). Wounds within a radiation-exposed area are associated with chronic pain. Such wounds are often refractory to reconstructive surgery or skin grafts and are susceptible to chronic infections [13,14]. Wound healing is a complex process that requires balance between inflammation, cell proliferation, and remodeling processes that can include fibroblast recruitment to the laceration site [17,18]. Once recruited, these fibroblasts differentiate into myofibroblasts that express various extracellular matrix (ECM) proteins that assist in proper wound closure and repair. Once the wound is closed, these myofibroblasts normally undergo apoptosis, preventing the over-production of scar-tissue-related ECMs. However, myofibroblasts in areas of radiation exposure fail to enter apoptosis and instead become activated, resulting in the overexpression of fibrosis-related ECM proteins, resulting in increased stiffness and thickening of the skin [19]. Unfortunately, there are no accepted medical treatments approved by the U.S. Food and Drug Administration (FDA) for the prevention or mitigation of radiation dermatitis or combined injuries [8,12,20,21,22].

Studies indicate that in humans ~2 Gy radiation exposures can induce moderate radiation dermatitis, and exposures of ≥12–18 Gy cause severe radiation dermatitis [12,20,23]. Exposure to single high dose or fractionated radiation > 20–25 Gy in humans can result in areas of non-healing radiation dermatitis [8,12,21,23,24]. Similar dose ranges have been reported in historical accidental radiation exposures and in survivors of the Hiroshima and Nagasaki atomic bombings [8]. Although visibly similar, radiation burns are pathophysiologically distinct from electrical or thermal burns [12,20,25,26,27]. All of these burns exhibit erythema, dry or moist desquamation, ulceration, and necrosis [20,28,29]. Defining characteristics of radiation dermatitis are inflammation outside of the radiation field, the loss of adult stem cells, and the development of opiate-resistant chronic pain [12]. Exposure of the skin to radiation results in a variety of mechanisms of cell death, including apoptosis and ferroptosis, and can induce accelerated senescence, also known as stress-induced premature senescence (SIPS) [2,24,30,31,32,33,34]. Accelerated senescence, a dominant radiation response of normal (non-transformed, non-immortalized) cells to radiation, has been associated with the loss of adult skin stem cells, increased chronic inflammation, altered cell–cell contacts, and ongoing tissue repair failure [14,31,32,34,35,36,37].

Our laboratory and others demonstrated that genistein, a naturally occurring, multifunctional isoflavone derived from soybeans, has significant prophylactic activity as a radiation countermeasure [38,39,40,41]. Genistein binds estrogen receptors as well as several kinase receptors, resulting in the modulation of a wide variety of signaling pathways [42,43]. Both clinical and preclinical studies have reported that genistein can act as an antioxidant, anti-inflammatory, antimicrobial, and antiviral agent, and it has also been attributed with chemotherapeutic properties [42,43]. Research from our laboratory and others has shown that genistein protects the bone marrow, lung, and intestine from acute and delayed radiation injuries [38,40,44,45]. However, the efficacy of genistein in preventing radiation dermatitis or CI has yet to be explored.

Due to the lack of U.S. FDA-approved countermeasures for radiation dermatitis, we sought to determine if prophylactic administration of genistein would reduce the severity of CRIs and/or improve healing of tissue in CI. Using our previously developed CRI murine model [24], C57BL/6 mice were sham irradiated or exposed to 16.9 Gy X-ray radiation within the thoracic region. Immediately after irradiation, a standardized dorsal full thickness skin wound was created within the irradiated field. This model enables investigation of delayed wound healing and non-healing radiation dermatitis. Although the irradiation of the thoracic region also results in lung injury, no early mortality is observed, and animals typically survive until ~180 days post-irradiation [46,47]. Wound closure and radiation dermatitis were monitored through 85 days following wound, CRIs, or CI. Genistein was not only able to prevent radiation-induced dermatitis and subsequent fibrosis but also improved the kinetics of wound closure in the CI model. Reduced radiation dermatitis was associated with a downregulation of cyclin-dependent kinase inhibitor 1 (p21/waf1) and improved survival of adult stem cells within the hair bulge region. Results suggest that genistein may be a promising prophylactic agent for radiation dermatitis.

## 2. Results

### 2.1. Comparison of General Health, Wound Closure, and Dermatitis in Three Murine Models

Although murine models exist for cutaneous radiation injury (CRI) and cutaneous radiation injury with wounding (termed combined injury, CI), these models have not been completely characterized or compared to each other to determine differences in cellular effects or pathophysiology. Our previously published dose–response for C57BL/6 mice showed that 14 Gy and lower doses exhibited spontaneous repair [24]. We subsequently administered 16–16.5 Gy, but the results in the animals were not uniform. Doses of 17.9–18.9 Gy resulted in severe radiation dermatitis with bleeding. The 16.9–17 Gy X-ray irradiation dose range resulted in non-healing radiation dermatitis in C57BL/6 mice that was not overly severe. This dose of radiation in the thoracic region does result in pulmonary injury at ~120–180 days, but there is no early mortality [46,47]. We compared general health, wound closure time, and dermatitis in sham-irradiated mice, a wound-only murine model, a CRI murine model, and a CI model. Previous studies from our laboratory and others demonstrated that genistein could be administered prophylactically to prevent radiation damage to multiple tissues [38,40,44,45]. Therefore, we also investigated the effects of prophylactic administration of genistein in the CI murine model.

**Figure 1:**
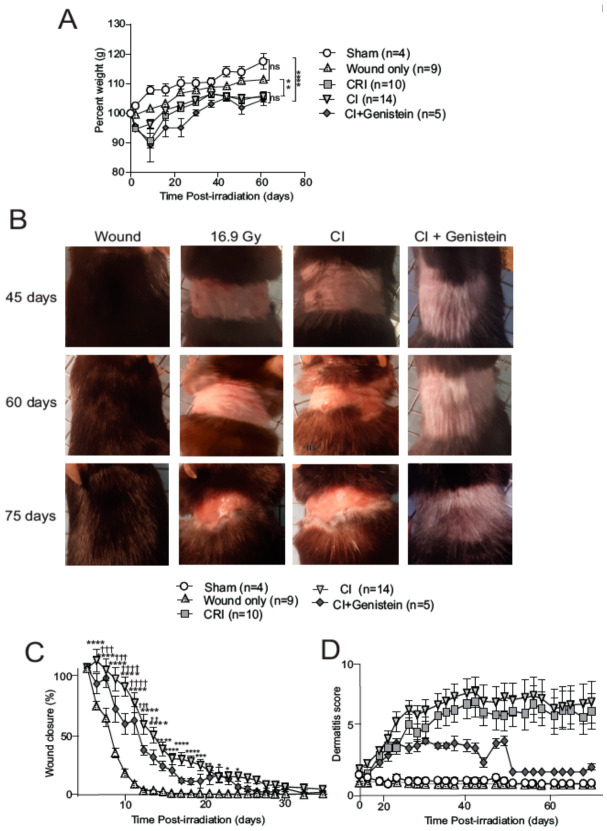
16.9 Gy X-ray irradiation induces radiation dermatitis and delays wound closure in a murine model of CI. C57BL/6 mice were divided into five groups: (1) sham irradiated (*n* = 4); (2) experimental wound (*n* = 9); (3) 16.9 Gy thoracic irradiation (CRI, *n* = 10); (4) thoracic irradiation and wounded (CI, *n* = 14); or (5) CI with genistein treatment (200 mg/kg, s.c.) 24 h before irradiation. (**A**) Weights of animals were obtained over time as a measure of general health. (**B**) Photographic images of the thoracic regions of mice at the indicated time points. Representative images are shown. (**C**) Percent wound closure over time. Wounds were digitally imaged, and the wound area was measured over time. Difference between wound only (group 2) and CI (group 4), * *p* < 0.05; ** *p* < 0.01; *** *p* < 0.005; **** *p* < 0.001. Difference between CI (group 4) and CI + genistein (group 5), †† *p* < 0.01; ††† *p* < 0.005; †††† *p* < 0.001. (**D**) Radiation dermatitis was scored over time. Graphs show means ± SEM.

As a proxy for general health, the body weight was measured bi-weekly (Figure 1A). Sham mice (no radiation and no wound) exhibited an expected gradual increase in weight gain throughout the study. Mice that received an experimental wound without thoracic irradiation (wound only) exhibited a non-significant delay in weight gain ~10 days post-wounding but then gained weight throughout the study, resulting in no significant difference from the weight of the sham group. However, mice exposed to thoracic irradiation, irrespective of treatment or wounding status, all showed a rapid 5–10% decrease in body weight at 2 to 10 days post-irradiation, followed by a subsequently gradual increase in percent weight gain. Irradiated mice never regained weight comparable to non-irradiated counterparts (for all radiation groups, *p* < 0.01 compared with wound-only mice, and *p* < 0.001 compared with sham mice). In this pilot study, treatment with genistein did not significantly affect overall weight loss from thoracic irradiation.

Wound repair and radiation-induced dermatitis were visually evaluated throughout the study (Figure 1B–D). Mice were photographed daily to document the rate of wound closure (Figure 1B). The wounds of mice not exposed to radiation completely closed within ~14 days (Figure 1C). Radiation exposure impaired wound closure, causing a reduced rate of wound closure compared with non-irradiated mice (Figure 1C). In sham-irradiated animals, 95% wound closure occurred within 11 days. In contrast, 95% wound closure in the CI occurred at ~28 days post-wounding/irradiation (Figure 1C). In this pilot study, genistein significantly improved the early rate of wound closure in the CI model on days 6–13 (Figure 1C), although the time point of full wound closure was not significantly improved from CI without treatment.

Mice exposed to thoracic radiation, irrespective of wounding status, developed radiation dermatitis starting ~14 days post-irradiation, with themaximal dermatitis score 7.7 in CI and maximal dermatitis score 6.9 in CRI at 42 days post-irradiation (Figure 1D). Thereafter the dermatitis scores in CI and CRI fluctuated but remained above 5.6 and did not exhibit significant repair. Interestingly, despite an initial increase in dermatitis score ~14 days post-irradiation, mice treated with genistein showed a decline in dermatitis at 21 days post-irradiation. By 70 days post-irradiation, the average dermatitis score of mice treated with genistein was 1.4 (SEM = 0.24), lower than the CRI or CI groups. The genistein-treated animals appeared similar to sham-irradiated mice but with white hair. Note that administration of PEG vehicle did not improve radiation dermatitis in the CRI model and did not induce hair regrowth (Figure S1A,B).

### 2.2. Genistein Reduces Radiation-Induced Acanthosis and Immune Cell Infiltration

**Figure 2:**
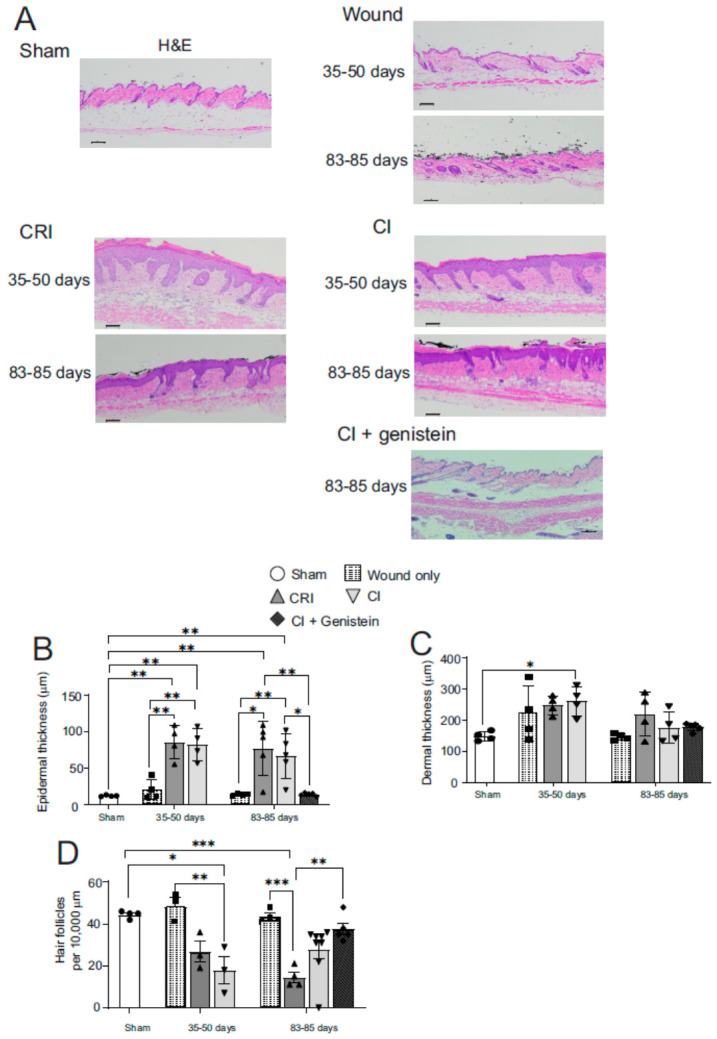
Structural alterations are induced by CRI and CI are prevented by genistein. C57BL/6 mice were divided into five groups: (1) sham irradiated; (2) experimental wound; (3) 16.9 Gy thoracic irradiation; (4) thoracic irradiation and wounded; or (5) CI with genistein treatment (200 mg/kg, s.c.) 24 h before irradiation, *n* = 4–5 animals per group. (**A**) Full thickness skin tissue was obtained at either 35–50 days or 83–85 days and used for H&E staining. Scar bar: 100 µm. Images show 20× magnification. Representative images are shown. Microscopic images were used to measure skin thickness as described in Section 4: (**B**) Epidermal thickness. (**C**) Dermal thickness. (**D**) Hair follicle counts. Graph show means ± SEM. * *p* < 0.05; ** *p* < 0.01; *** *p* < 0.001.

Our laboratory and others previously observed epidermal thickening from radiation exposure in C57BL/6 mice [24,48,49]. We examined histological alterations in skin structures with wound, CRI, and CI at early time points (35–50 days post injury/irradiation) and late time points (83–85 days post injury/irradiation), and we examined the effect of genistein at the late time point on CI (Figure 2). Hematoxylin and eosin (H&E) stained sections were used to measure epidermal and dermal thickness (Figure 2A, left panel). The presence of an experimental wound did not alter the average epidermal thickness at either early (20.16 ± 7.07 µm) or late (13.2 ± 0.8 µm) times compared to sham mice (11.4 ± 0.53 µm) (Figure 2B). However, in CRI and CI mice, there was a 6–10-fold thickening of the epidermis (acanthosis) at both time points (Figure 2A left panel B; *p* < 0.01 for both compared with sham). Mice treated with genistein had an epidermal thickness that was not significantly different from sham (13.5 ± 0.97 µm) but was significantly reduced from the radiation and radiation + wound groups (Figure 2B; *p* < 0.05 and 0.1, respectively). In the CI group, but not the CRI group, there was a statistically significant increase in dermal thickness at the early time point (260.24 ± 46.81 µm) compared to sham mice (148.21 ± 14.77 µm; *p* < 0.01) (Figure 2C). No statistical variation was observed in dermal thickness in the skin of CRI or CI mice during late dermatitis development, suggesting that dermal thickening was resolved. Dermal thickening in CRI with PEG vehicle treatment was similar to that observed in CRI (Figure S2B, left panel). Radiation, but not wounding alone, also caused a decrease in hair follicle density (Figure 2D). Data show that compared with the sham group, the decrease in hair follicles was significant at 35–50 days after CI and at 83–85 days after CRI (*p* < 0.05 and 0.005, respectively). The decrease in hair follicles in CI was not significantly different from sham at 83–85 days. In this pilot study, genistein treatment of mice in the CI group also resulted in a retention of hair follicles comparable with sham (Figure 2D).

**Figure 3:**
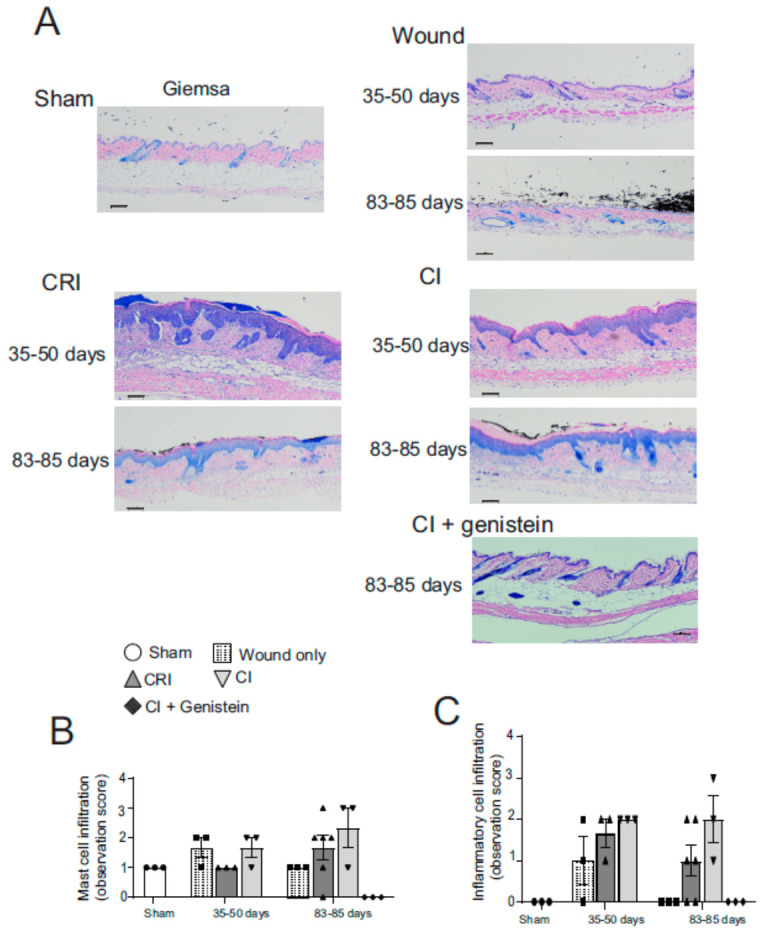
CRI and CI increase numbers of inflammatory cells that are reduced by genistein treatment. C57BL/6 mice were divided into five groups: (1) sham irradiated; (2) experimental wound; (3) 16.9 Gy thoracic irradiation; (4) thoracic irradiation and wounded; or (5) CI with genistein treatment (200 mg/kg, s.c.) 24 h before irradiation, *n* = 3–5 animals per group. (**A**) Full thickness skin tissue was obtained at either 35–50 days or 83–85 days and used for Giemsa staining for mast cells. Representative images are shown. Scar bar: 100 µm. Images show 20× magnification Microscopic images were used to score inflammatory cell infiltration as described in Section 4: (**B**) Mast cell infiltration. (**C**) Neutrophil and macrophage infiltration. Graphs show means ± SEM. Note that statistical analysis is not performed on the pathology scores.

Inflammation in the skin post-irradiation has been described by our laboratory and others [24,48,50]. We used Giemsa staining together with H&E staining to evaluate infiltration of inflammatory cells in the mouse skin at early and late time points and to determine the effect of genistein treatment (Figure 3). Evaluation of Giemsa staining suggested that there was a slight increase in mast cell infiltration, predominantly in the dermis (Figure 3A,B and Figure S1). However, in both CRI and CI groups in the irradiated regions, particularly surrounding ulcers, there was a marked infiltration of both neutrophils and macrophages in the dermis (Figure 3B and Figure S1). Genistein treatment in this pilot study appeared to reduce the levels of neutrophils and macrophages in the dermis to baseline levels (Figure 3C). Note that inflammation observed in CRI with PEG vehicle treatment was similar to CRI (Figure S2).

### 2.3. Genistein Prevents Radiation-Induced Fibrotic Remodeling

**Figure 4:**
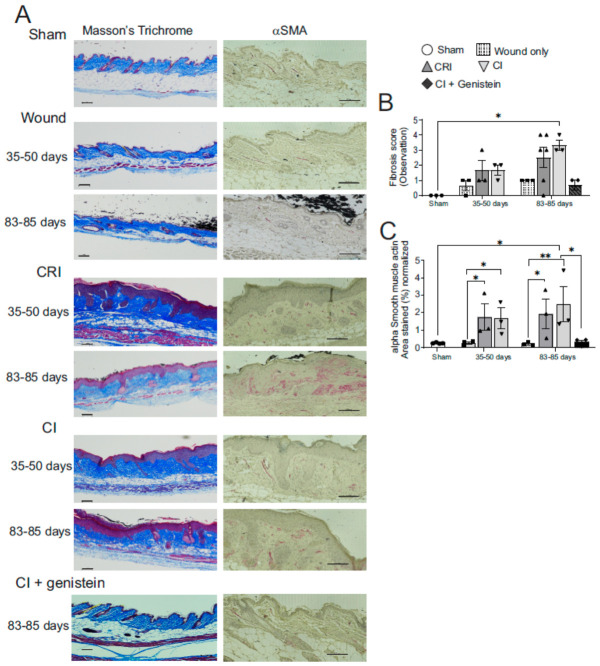
CRI and CI result in fibrotic remodeling that is prevented by genistein treatment. C57BL/6 mice were divided into five groups: (1) sham irradiated; (2) experimental wound; (3) 16.9 Gy thoracic irradiation; (4) thoracic irradiation and wounded; or (5) CI with genistein treatment (200 mg/kg, s.c.) 24 h before irradiation, *n* = 3–5 animals per group. (**A**) Full thickness skin tissue was obtained at either 35–50 days or 83–85 days and used for Masson’s Trichrome staining (left panel) or alpha smooth muscle actin (αSMA) immunohistochemistry (right panel). Representative images are shown. Scar bar: 100 µm. (**B**) Microscopic images were used to score fibrosis by a pathologist as described in the Section 4. (**C**) αSMA quantification, as described in Section 4. Graphs show means ± SEM. For αSMA graph * *p* < 0.05; ** *p* < 0.01.

In addition to an inflammatory response, radiation induces fibrosis, an additional factor that affects wound healing and dermal structure and function [19,24,51,52]. Masson’s trichrome stain was used to detect collagen deposition (Figure 4A, left panel). To quantitatively evaluate the amount of fibrosis, we evaluated the level of alpha-smooth muscle actin (α-SMA) expression, a known marker of fibrotic remodeling, by immunohistochemistry (Figure 4A, left panel). The Masson’s trichrome staining for collagen deposition, as well as structural alterations in the skin, was evaluated and scored from none (grade 0) to severe (grade 5) by a pathologist blinded to the treatment groups. A slight increase in fibrosis was observed in wounded mice compared to sham in both in the late early and lateearly dermatitis time points. Histological scoring of fibrosis showed a non-significant 1.5–2-fold increase in fibrosis score in both CRI and CI models at 35–50 days, and 2.5–3-fold in fibrosis score at 83–85 days. The only significance for fibrosis scoring was in the CI group at 83–85 days (*p* < 0.05; Figure 4B). The fibrosis score of genistein-treated CRI animals was not different from sham (Figure 4B). Note that PEG treatment in the CRI model did not significantly alter the level of fibrosis from CRI (Figure S2B, right panel).

We quantified the increased α-SMA, a form of actin expressed in activated myofibroblasts, as a marker of fibrotic remodeling. Increased expression of α-SMA occurred primarily in the dermis (Figure 4A, right panel; quantification Figure 4C). Interestingly, wounding alone did not result in increased α-SMA. CRI resulted in non-significant increases in α-SMA at both the early and late time points, but CI resulted in a significant increase at both time points (~4-fold 35–50 days, *p* < 0.05; ~8-fold at 83–85 days, *p* < 0.05). Treatment with genistein resulted in α-SMA levels and organization that were not different from sham.

### 2.4. Genistein Treatment Preserves Adult Skin Stem Cells and Prevents Hyperproliferation of Epidermis Keratinocytes

**Figure 5:**
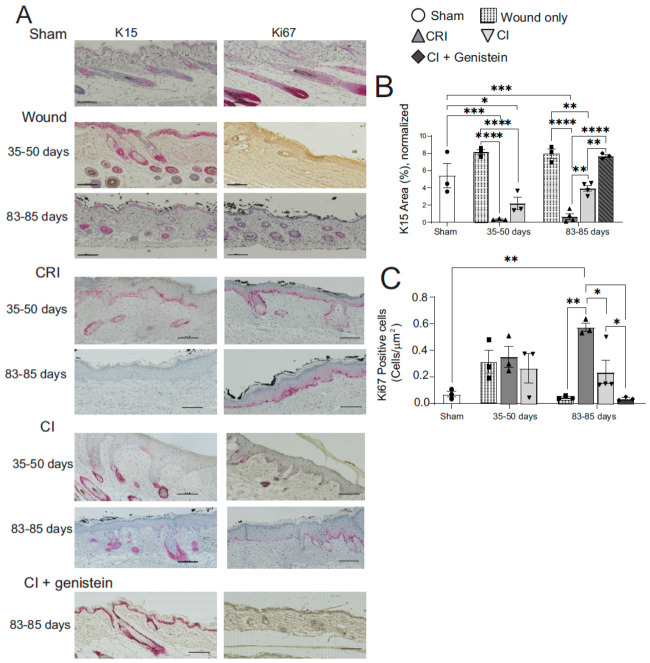
CRI and CI result in reduced stem cells but increased proliferation of epithelial keratinocytes that are prevented by genistein treatment. C57BL/6 mice were divided into five groups: (1) sham irradiated; (2) experimental wound; (3) 16.9 Gy thoracic irradiation; (4) thoracic irradiation and wounded; or (5) CI with genistein treatment (200 mg/kg, s.c.) 24 h before irradiation, *n* = 3–5 animals per group. (**A**) Full thickness skin tissue was obtained at either 35–50 days or 83–85 days and used for IHC for K15 (stem cell marker, left panel) or Ki67 (proliferation marker, right panel). Representative images are shown. Scar bar: 100 µm. (**B**) Microscopic images were used to score the percent of K15 stained area using ImageJ, as described in Section 4. (**C**) Ki67 quantification was performed using ImageJ, as described in Section 4. Graphs show means ± SEM. For Ki67 graph * *p* < 0.05; ** *p* < 0.01; *** *p* < 0.001; **** *p* < 0.0001.

We previously demonstrated that radiation induces a loss of skin adult stem cells [24]. Interestingly, the simultaneous thickening of the surface layer of keratinocytes suggests that there is exaggerated cellular proliferation in cellular populations in the top-most layer of cells [24]. We therefore evalulated adult skin cell populations and cellular proliferation at the early and late time points after wounding, CRI, and CI (Figure 5). Immunohistochemistry of K15 (marker for keratinocytes and adult stem cells) showed strong staining in the bulge region of the hair follicle, with lighter staining in the epithelial layer, in sham-irradiated mice and in mice after wounding (Figure 5A, left panel; quantification Figure 5B). Wounding alone did not reduce the level of K15 staining in the hair bulge region compared with sham. At 35–50 days and at 83–85 days post-CRI, K15 staining was significantly reduced in the hair bulge region (*p* < 0.005 compared with sham for both time points). In the CI model, K15 was significantly reduced in the hair bulge regions at 35–50 days compared with sham (*p* < 0.05) but showed a trend toward recovering at 83–85 days, suggesting that the presence of the wound mitigated the loss of the adult stem cells. Mice receiving prophylactic genistein treatment retained K15 staining, with a staining pattern indistinguishable from sham and wounded mice. K15 staining was significantly improved by genistein treatment compared with CI + vehicle at 83–85 days (*p* < 0.01).

We quantified cellular proliferation (Ki67 stained cells) in the epithelial layer as a measure of keratinocyte proliferation (Figure 5A, right panel; quantification Figure 5C). Quantification was performed for proliferative cells in the epithelium. Sham mice showed a low level of Ki67 staining in the epithelial layer, suggesting the presence of a low level of keratinocyte proliferation under homeostatic conditions. In the wound-only group, at 35–50 days, there was a ~3-fold increase in Ki67 staining, but this did not reach significance compared with the sham group. Ki67 staining returned to baseline in this group by 83–85 days. CRI mice showed a non-significant increase, ~3-fold higher than baseline, at 35–50 days, but demonstrated a ~6-fold increase at 83–85 days (*p* < 0.01). In the CI model, there was a non-significant trend of increased Ki67 staining compared with the sham group at both the early and late time points. Genistein treatment in this pilot study reduced the number of proliferating keratinocytes compared to both the CRI and CI models at 83–85 days (*p* < 0.005 and <0.05, respectively), and the level of Ki67 staining was not different from the sham group.

### 2.5. Genistein Alters Radiation-Induced Accelerated Senescence and Apoptosis

**Figure 6:**
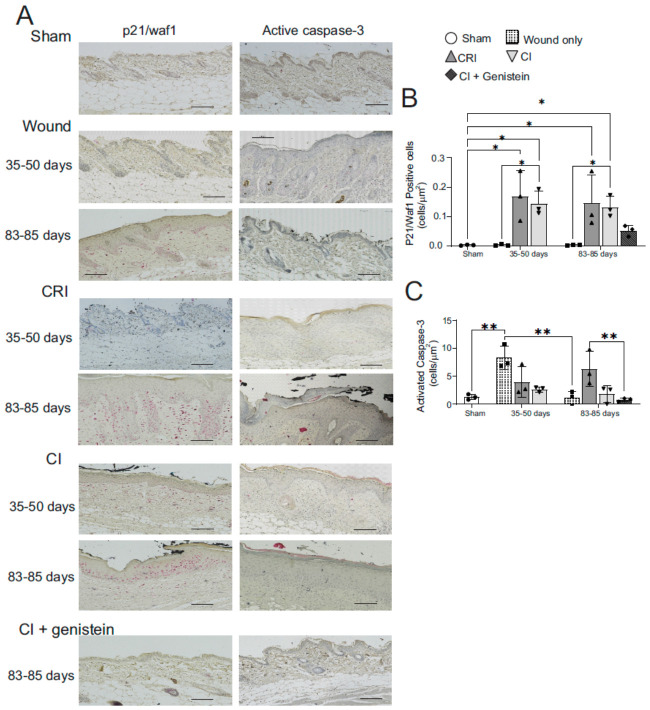
CRI and CI result in increased accelerated senescence and apoptosis that are reduced by genistein treatment. C57BL/6 mice were divided into five groups: (1) sham irradiated; (2) experimental wound; (3) 16.9 Gy thoracic irradiation; (4) thoracic irradiation and wounded; or (5) CI with genistein treatment (200 mg/kg, s.c.) 24 h before irradiation, *n* = 3–5 animals per group. (**A**) Full thickness skin tissue was obtained at either 35–50 days or 83–85 days and used for IHC for p21/waf1 (accelerated senescence marker, left panel) or activated caspase-3 (apoptosis marker, right panel). Representative images are shown. Scar bar: 100 µm. (**B**) Microscopic images were used to quantify p21/waf1, as described in Section 4. (**C**) Microscopic images were used to quantify activated caspase-3, as described in Section 4. Graphs show means ± SEM; * *p* < 0.05; ** *p* < 0.01.

Our laboratory and others showed that high dose irradiation induces both apoptosis and senescence in the skin [24,35,53,54,55]. We investigated apoptosis and accelerated senescence in our models (Figure 6). Wounding did not significantly increase cyclin-dependent kinase inhibitor 1 (p21/waf1, marker for senescence) at either time point (Figure 6A, left panel; quantified in Figure 6B). However, radiation exposure in CRI and CI models resulted in ~15–20-fold increase in p21/waf1 levels at both the early and late time points (*p* < 0.01). However, the level of p21/waf1 in genistein-treated mice was not statistically distinct from sham mice. Activated caspase-3 IHC was used to evaluate apoptosis in the tissues.

Caspase-3 expression was significantly increased, ~7-fold, in wounded mice at 35–50 days (*p* < 0.01), which returned to basal levels once repaired. CRI and CI mice showed non-significant increases in caspase-3 at both the early and late time points (~3–5-fold). Genistein treatment of CI resulted in significantly less activated caspase-3 compared to CRI but was not significantly reduced compared with CI (Figure 6C, *p* < 0.01).

## 3. Discussion

The treatment of radiation burns, both accidental and arising from clinical radiation exposures, remains a medical challenge [20,56,57]. Historically, human radiation burns are characterized by erythema, desquamation, inflammation, and necrosis, often in conjunction with failed cycles of attempted tissue repair [20,56,57]. In this study we compared and characterized murine models of wound alone, cutaneous radiation injury (CRI), and combined injury (CI) with wound and radiation injury. Our data show that the wound-only model was characterized by increases in inflammation and apoptosis at 35–50 days that were resolved by 83–85 days. The CRI model displayed radiation dermatitis characterized by sustained increased inflammation, epithelial thickening, adnexal loss with loss of skin stem cells, long-term accelerated senescence, and fibrotic remodeling. In contrast, the CI model exhibited sustained radiation dermatitis with epithelial thickening, inflammation, adnexal loss but with retention of some skin stem cells, long-term accelerated senescence, and fibrotic remodeling. Furthermore, our data show that prophylactic administration of genistein in the CI model resolved most of the pathological effects of radiation on the skin. Together, our data show key differences between the CRI and CI models and suggest that genistein treatment can prevent radiation-induced changes in the CI model.

In this pilot study, we compared wound only to both CRI and CI. The wound-only group differed in several ways from the two irradiated groups. Wound-only tissues fully recovered skin structures such as hair follicles and sebaceous glands by ~35 days post-wounding, and there was no significant increase in epithelial or dermal thickening at either time point. At the 35–50-day time point, the wounded skin did exhibit a transient increase in neutrophil and macrophage infiltration that resolved by 83–85 days. The wounded tissues also displayed increased activated caspase-3 and increased keratinocyte proliferation at the early time point that also later resolved. Previous studies have demonstrated caspase-3’s role in stimulating the proliferation of cells within the wound periphery to promote wound healing [58]. Although wounded tissues did display a minimal increase in fibrosis scoring, this was not accompanied by a persistent increase in cells expressing α-SMA. Finally, wounded tissues did not display any increase in p21/waf1 expression at any time point, suggesting that accelerated senescence signaling was not activated.

Our pilot study revealed that the CRI and CI models were similar in many respects. The most notable differences between the CRI and CI models were the persistent loss of normal skin structures, including the loss of hair follicles and sebaceous glands, with persistent thickening of the epithelial layer (~6–7-fold increase in both models compared with control). Only the CI model displayed significant increases in the thickness of the dermal layer (~60% increase), but this occurred only at the early time point and was resolved by 83–85 days. Thickening of the epidermis was concurrent with increased proliferation of the epithelial keratinocytes, with ~5–10-fold increase in the expression of the proliferation marker Ki67 in this cell population. The fibrosis pathology scores in both the CRI and CI models were minimal to mild at the early time point but increased in both models to moderate-marked at the later time point. At both time points in both models, the number of cells expressing α-SMA could be associated with the increased fibrosis scores. Both the CRI and CI models also displayed increased numbers of inflammatory cells. Interestingly, although there was a trend toward increased mast cells in the tissues in both CRI and CI, most of the scored increase in inflammation was due to increased numbers of neutrophils and macrophages. Finally, both the CRI and CI models displayed significant increases in p21/waf1 expression, suggesting chronic accelerated senescence was present in the tissues. Overall, the loss of normal skin structures and chronic fibrosis, inflammation, and senescence are consistent with radiation-induced injuries that have been demonstrated in other tissues.

There were several differences between the CRI and CI models. Compared with the CRI tissues, the tissue from CI mice displayed some retention of adult stem cells, as indicated by K15 staining compared with CRI at 83–85 days. Consistent with this, there was also a higher presence of follicle-like structures in CI compared with CRI, although there was still a lack of visible hair at the time of euthanasia. Additionally, the CRI model displayed increased caspase-3 activation at the 83–85-day time point, compared to little or no caspase-3 activation in the CI model, suggesting that chronic apoptosis occurs in CRI but not CI. This suggests that chronic apoptosis may be contributing to the sustained loss of adult stem cells. The mechanism by which a wound within a radiation field activates anti-apoptotic activity is not known. Further work is needed to better understand how the CRI and CI models differ in the types of cellular damage that occur.

The development of prophylactic agents for radiation dermatitis has received significant attention for both military and civilian uses. The Department of War (DoW) must maintain mission capacity even after radiation exposure events. The U.S. military also provides first responder and humanitarian aid, as seen following the 2011 Fukushima nuclear plant disaster (Operation Tomodachi) [59]. In this crisis, Uniformed Services University of the Health Sciences (USU), under the DoW, provided critical radiation expertise and led radiation monitoring [59]. Since the terrorist events of 11 September 2001, the US Government has allocated almost $1 billion for medical countermeasures (MCM) development to ensure medical preparedness for a nuclear and/or radiological incident, including for the development of prophylactic agents. Recently, the Russian invasion of Ukraine threatened radiation exposure through potential tactical nuclear weapon strikes and attacks on nuclear facilities, such as the Zaporizhzhia power plant. Radiotherapy for the treatment of cancer also provides another scenario for the use of prophylactic agents for radiation dermatitis, although agents would undergo an additional test to ensure that they did not protect the targeted cancer [12].

The development of prophylactic agents to prevent radiation dermatitis is an important goal for the military and first responders who may be required to enter areas of radiation contamination. Additionally, such agents may be useful for protecting normal tissues in patients undergoing radiotherapy for cancer treatments if they do not protect the cancerous tissue itself. Previous studies, including studies from our laboratory, showed that the soy isoflavone genistein is a potent radiation countermeasure when administered prophylactically [38,39,40]. Our previous data suggested genistein treatment induced stem cell quiescence in the bone marrow followed by reduced senescence that was associated with reduced DNA damage and the prevention of acute myelotoxicity in a total body irradiation model [38,40]. We also found that prophylactic genistein treatment reduced DNA damage in the lung in a murine model of radiation-induced delayed lung injury [39,47]. Our current data show that genistein administered by subcutaneous injection 24 h before radiation exposure significantly prevented radiation dermatitis and fibrotic remodeling in the murine CI model. Genistein also improved the early rate of closure of the wound but did not significantly change the final time point of wound closure. This could be significant for patients, as the early period of wound closure is the time that is most susceptible to bacterial and fungal infection. Importantly, genistein reduced epithelial thickening, inflammation, fibrotic remodeling, and accelerated senescence in the murine CI model. Genistein-treated tissues had a marked improvement in normal skin structures, including increased stem cells and hair follicles. Together, our data suggest that the prophylactic activity of genistein likely extends to skin tissues; our findings lay the groundwork for a larger investigation of the effects and mechanism of genistein on radiation dermatitis.

## 4. Materials and Methods

### 4.1. Reagents

Unless otherwise noted, chemicals were purchased from Sigma-Aldrich, St. Louis, MO, USA.

### 4.2. Animals

All animal experiments were approved by the Uniformed Services University of the Health Sciences (USU) ethics committee Institutional Animal Care and Use Committee (IACUC) (protocol PHA-21-045, approved 4 April 2021) and were conducted in compliance with the Animal Welfare Act and in accordance with the principles in the “Guide for the Care and Use of Laboratory Animals” [60]. Female C57BL/6 mice were purchased from Jackson Laboratories (Bar Harbor, ME, USA). Mice were housed in groups of four or five, in rooms maintained at 21 ± 2 °C, 50% ± 10% humidity, and a 12 h light/dark cycle in the USU animal facility, accredited by the Association for Assessment and Accreditation of Laboratory Animal Care International. Commercial rodent ration (Harlan Teklad Global 18% Protein Rodent Diet 8604, Harlan Laboratories, Madison, WI, USA) and acidified water (pH = 2.5–3.0, to control opportunistic infections) were freely available [61].

### 4.3. Thoracic Irradiation, Experimental Wounds, Genistein Administration, Scoring, and Treatment for Radiation Dermatitis

**Figure 7:**
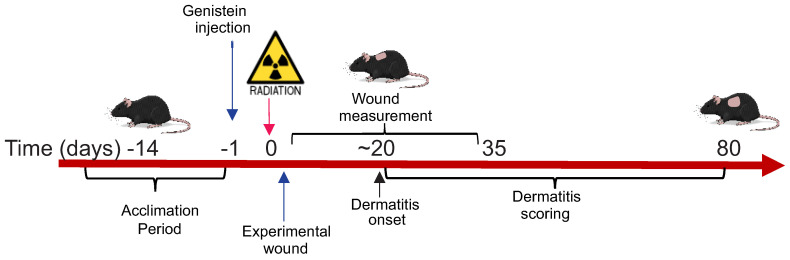
Schematic of experimental protocol for radiation dermatitis and combined injury. After acclimation (14 days), mice were divided into five groups: (1) sham irradiated; (2) experimental wound; (3) 16.9 Gy thoracic irradiation; (4) thoracic irradiation and wounded; or (5) CI with genistein treatment (200 mg/kg, s.c.) 24 h before irradiation. The onset of dermatitis occurred approximately 2–4 weeks after radiation exposure. Mice were visually evaluated every 3–4 days to score dermatitis (hair loss, erythema, scale, and ulceration) and to measure wound closure. After 35–85 days, mice were euthanized and skin samples were collected for histological and immunohistochemical evaluation.

Animals were randomized into the different treatment groups. Experiments were performed with two control groups that received sham irradiation and no wound (Sham) and wound only and no irradiation (Wound only) (Figure 7). X-ray irradiation was performed using an RS2000 Biological Research Irradiator (Rad Source Technologies, Suwanee, GA, USA) as previously described [24]. Irradiation was performed with 160 kVp, 25 mA, and 0.3 mm Cu beam filtration. A lead shield was used to allow thoracic radiation exposure while shielding the rest of the body [24]. The lead shield was placed on the floor of the irradiator; the mouse’s thorax was ~43.75 cm below the X-ray source. Dosimetry was performed using cylindrical acrylic mouse phantoms provided by the University of Wisconsin Medical Radiation Research Center (UW MRRC), each embedded with three (1 × 1 × 1 mm) Harshaw TLD-100 microcubes (Thermo Electron Corp., Oakwood Village, OH, USA). TLDs were processed at UW MRRC using a national standard with an expanded uncertainty (k = 2) of 5%. The dose rate for each aperture position was reported as an absorbed dose rate to water (ADRW, Gy/min), and two repeated measurements were averaged. The average ADRW at any position was 0.775 Gy/min with 97% uniformity and TLD measurement had an expanded uncertainty of ±5.2%. Two days prior to irradiation, mice (12–14 weeks of age) were anesthetized (intraperitoneal injections of 150 mg/kg ketamine, 18 mg/kg xylazine), and the thoracic regions were shaved on the posterior and sides. On the day of irradiation, mice were anesthetized and irradiated in the prone position in Lucite jigs (3 mm thick) to prevent movement. Immediately following irradiation, wounds were administered within the irradiated site. Prior to wounding, the dorsal skin of mice was sterilized by the alternating application of iodine solution and 70% ethanol from the center outward to maintain aseptic technique. The dorsal skin was gently lifted to form a fold, and a disposable 8 mm sterile biopsy punch was used to create a single circular wound within the irradiation field. Anesthetized animals were allowed to recover on a warming pad prior to returning to their original cages. Genistein was prepared as previously described [24,40,44]. Genistein was solubilized in polyethylene glycol, MW 400 (PEG 400), and sonicated for ~20 s prior to injection. Genistein was administered by a 100 µL subcutaneous injection, containing 200 mg/kg genistein in the area between the scapulae (above the area of irradiation) 24 h prior to irradiation. Vehicle-treated animals received PEG 400 subcutaneous injections with the same volume as genistein-treated animals. Individuals performing injections were blinded to the treatments. For each experiment, the *n* is provided in the figure legend. The order of treatments and measurements was performed according to a standard protocol to reduce potential confounders. The region of thoracic irradiation was scored from dermatitis onset (14 days post-irradiation) to the end of the study for hair loss, erythema (redness), desquamation (scaling), and ulceration using a modified scale 0–4 ranging from normal to severe for each criterion, as previously described [12,24,51]. Individuals scoring animals were blinded to the treatment groups. Total scores for radiation dermatitis were the sum of scores of each of the criteria. All animals displaying radiation dermatitis were treated daily using Silvadene and topical antibiotics as needed. Silvadene and antibiotic treatment did not alter radiation dermatitis scores but were administered for the care of the animals.

### 4.4. Tissue Sections, Skin Layer Measurements, and Inflammation and Fibrosis Scoring

Mice were euthanized at indicated time points, and full thickness skin, including the panniculus carnosus, was removed from the dorsal region, including cranial and caudal portions of the sections outside of the irradiated area. Tissues were fixed for 24 h in 10% neutral buffered formalin followed by 70% ethanol. Tissues were paraffin wax-embedded, sectioned at ~5 µm. Sections were then stained with hematoxylin and eosin (H&E), Masson’s Trichrome, or Giemsa [62]. Histological sections were scored for degree of mixed inflammatory cell (neutrophil and macrophage) and mast cell infiltration as well as fibrosis by a dermatology pathologist and a veterinary pathologist blinded to the treatment groups using the scoring lexicon: 0 = none; 1 = minimal; 2 = mild; 3 = moderate; 4 = marked; 5 = severe [63]. To determine an accurate average epidermal and dermal thickness for each section, the specific skin layer was measured every ~500–1000 µM throughout the irradiated field (~10,000 µM); rete ridges were not included in the measurements. Scoring for K15 staining in hair bulge regions for quantification of skin stem cells was performed by two dermatologists blinded to the treatment groups using score 2 = normal (sham), 1 = reduced from normal, 0 = absent, or 3 = greater than normal.

### 4.5. Wound Measurement

To quantitatively assess progression of wound closure, digital images of the wound were taken throughout the study. Mice were placed on a wire mesh platform and prompted to fully extend to enhance wound visibility. To ensure a consistent distance and angle between all images, a digital camera was mounted on a dunnage rack. A ruler was placed adjacent to the mouse to serve as a scale for the image to be calculated during analysis. The area of the wound was measured using the open-source Fiji ImageJ software version 1.54p (https://imagej.net/software/fiji/ accessed 25 May 2025) [64]. Once uploaded into ImageJ, the included ruler was used to set the scale in pixels per millimeter. The wound boundary was traced manually using the Polygon Selection tool, and wound area was automatically calculated (mm^2^). The percentage of wound closure at each time point was calculated relative to the wound size 5 days post wounding to primarily measure the effect of re-epithelization while minimizing the effect of contraction [65].

### 4.6. Immunohistochemistry

Formalin-fixed, paraffin-embedded (FFPE) mouse skin sections (4–5 µm) were used for immunohistochemistry. Sections were deparaffinized in xylene, rehydrated through a graded ethanol series (100%, 95%, 70%, phosphate buffered saline [PBS]), and subjected to antigen retrieval as specified for each primary antibody. Individuals performing IHC were blinded to the treatment groups. Sections were blocked with 10% normal goat serum (NGS) in PBS/0.1% Triton X-100 for 1 h at room temperature, then incubated overnight at 4 °C with primary antibodies diluted in 5% NGS PBS/0.1% Triton X-100/0.1% ultra-pure bovine serum albumin. Specific primary antibody staining methods: rabbit anti-K15 (ab52816, Abcam, Cambridge, MA, USA), antigen retrieval in 10 mM citrate buffer (pH 6.0) at 95 °C 20 min, slow cooling to room temperature, diluted 1:750 for staining; rabbit anti-p21/waf1 (ab188224, Abcam), antigen retrieval in 1 mM EDTA, 10 mM Tris (pH 9.0) at 95 °C 20 min, diluted 1:100 for staining; rabbit anti-cleaved caspase-3 (#9661S; Cell Signaling Technology, Danvers, MA, USA), retrieval 10 mM citrate buffer (pH 6.0) at 95 °C for 20 min, followed by cooling for 30 min, diluted 1:250 for staining but using 2% NGS; rabbit anti-Ki67 (#12202; Cell Signaling Technology), retrieval in 10 mM citrate buffer (pH 6.0) at 95 °C for 20 min, followed by cooling for 20 min., diluted 1:200 for staining; anti-rabbit α-SMA (#19245S Cell Signaling Technology) retrieval in 10 mM citrate buffer (pH 6.0) at 95 °C for 20 min, followed by cooling for 30 min, diluted 1:300 for staining. Slides were washed 2× incubated with biotinylated Goat Anti-Rabbit IgG (H+L) secondary antibody (BA-1000-1.5; Vector Laboratoiries), followed by detection using an avidin–biotin complex (ABC) alkaline phosphatase kit and visualization with ImmPACT^®^ Vector^®^ Red Substrate, alkaline phosphatase, for 18–20 min. (SK-5105; Vector Laboratoiries, Newark CA, USA). Sections were counterstained with Mayer’s hematoxylin or methylene blue (Kwik-Diff, Richard Allan Scientific, Kalamazoo, MI, USA), dehydrated, cleared in xylene, and mounted with a permanent mounting medium.

### 4.7. Quantification and Statistical Analysis

For analysis of body weight and wound closure, the total data collection was analyzed using Prism with two-way ANOVA. Immunohistochemistry quantification was performed using Fiji (Fiji Is Just ImageJ) ImageJ software, version 1.54p (https://imagej.net/software/fiji/ accessed on 25 May 2025) (NIH, Bethesda, MD, USA and, University of Wisconsin, Madison, WI, USA). For p21/waf1, Ki67, and cleaved caspase-3, the number of positive cells was counted and expressed relative to tissue area, averaged from three randomized, blinded images per animal (n = 3 per group). For α-SMA and K15, mean staining intensity and area were measured in Fiji under the same conditions. Statistical analysis was performed using GraphPad Prism 10 (GraphPad Software, La Jolla, CA, USA). Groups were compared by one-way ANOVA with Tukey’s post-test, and results are presented as mean ± SEM; *p* < 0.05 was considered statistically significant. Mast cell infiltration, inflammatory cell infiltration, and fibrosis scoring were presented as mean ± SEM and were statistically analyzed using a Kruskal–Wallis one-way ANOVA with a Dunn post hoc test.

## Data Availability

The original contributions presented in this study are included in the article/Supplementary Materials. Further inquiries can be directed to the corresponding author.

## Supplementary Materials

The following supporting information can be downloaded at: https://www.mdpi.com/article/10.3390/ijms27115019/s1.
