# Supplementary material for: Murine Model of Radiation Dermatitis with Experimental Wound and Effects of Genistein
Source: Int J Mol Sci. 2026 Jun 2;27(11):5019. doi: 10.3390/ijms27115019 (PMC13257261; doi:10.3390/ijms27115019)
Supplement: Supplementary file 1 [file ijms-27-05019-s001.zip › ijms-4294353-supplementary.pdf]

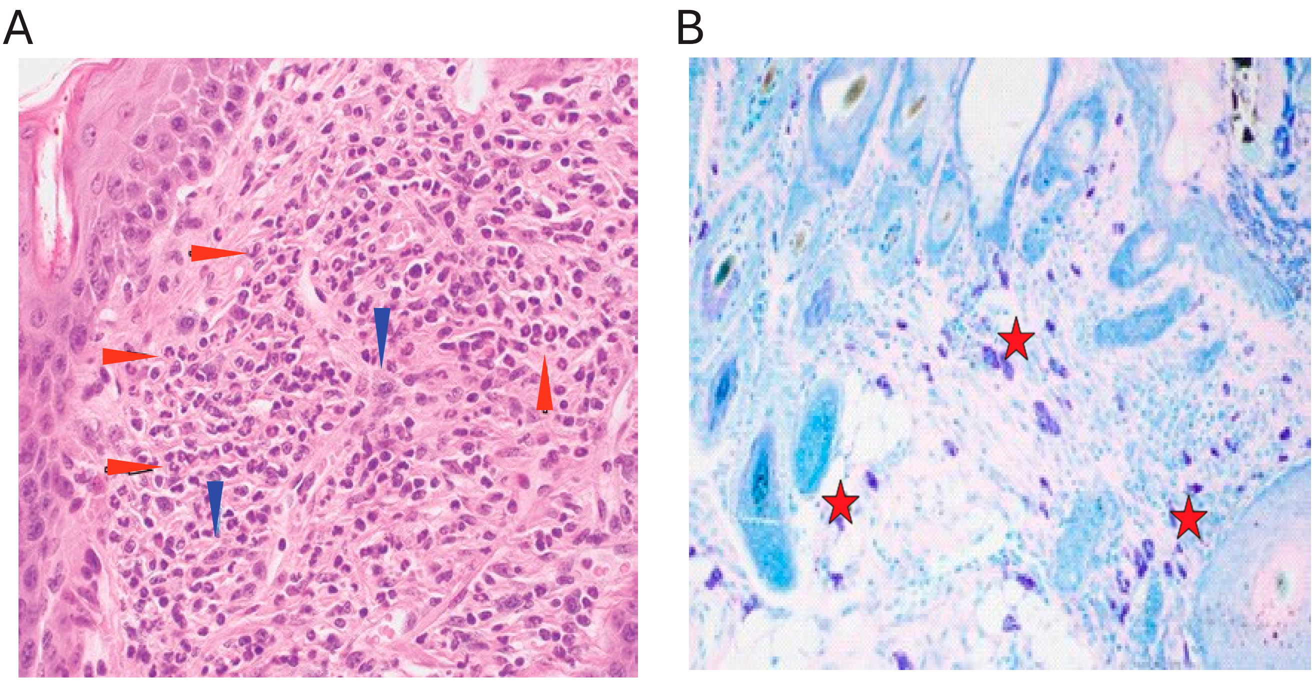

Figure S1. Inflammation in the skin of C57BL/6 mice in CI at 83 days post-irradiation. C57BL/6 female mice, 12-14 weeks of age, were exposed to 16.9 Gy thoracic X-ray irradiation and then received an experimental wound in the radiation field. Tissue was obtained at 83 days post-irradiation and used for histology. Tissue was stained for A. Hematoxylin & eosin or B. Giemsa. A.Tissue showed infiltration of mostly neutrophils (red arrows) with a lesser amount of macrophages (blue arrows; 400x magnification). B. Giemsa staining showed a moderate number of mast cells expanding the dermis (red stars; 200x magnification).

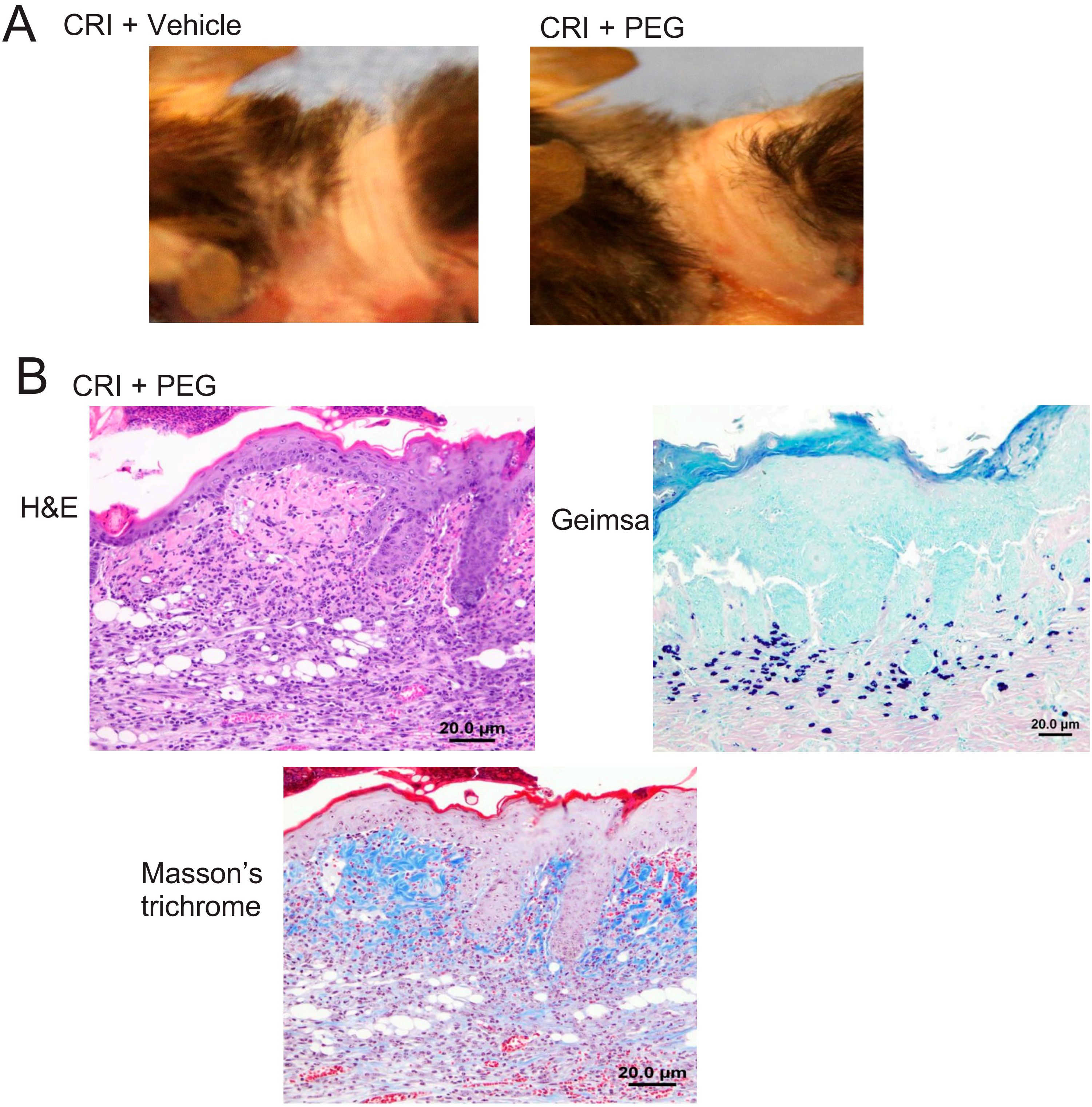

Figure S2. PEG-400 does not prevent radiation dermatitis in the C57BL/6 model. C57BL/6 female mice, 12-14 weeks of age, were injected with 100 ml of PEG-400 24 h prior to exposure to 16.9 Gy thoracic X-ray irradiation. A. Gross histology of uninjected mice at 80 days post-irradiation showing cutaneous radiation injury (CRI) + vehicle and CRI + PEG. B. Mice were treated with PEG-400 24 h prior to radiation exposure. Tissue was obtained at 83 days post-irradiation and used for histology. Tissue was stained for Hematoxylin & eosin (H&E), Giemsa, or Masson's Trichrome. Representative images are shown for N=3 individual mice. Microscopy shows 20x magnification. Note that the Masson's Trichrome staining was performed at the AFRRI histology laboratory, resulting in collagen staining a light grey.
